# Tandem receiver domain response regulator DrdR modulates swarming by controlling PilB/PilT activity in *Xanthomonas campestris*

**DOI:** 10.1002/mlf2.70092

**Published:** 2026-08-25

**Authors:** Peidong Ren, Jianrou Meng, Qijian Qin, Jiafeng Shi, Jiali Yao, Yanhua Qi, Jiliang Tang, Rui‐Fang Li, Guang‐Tao Lu

**Affiliations:** ^1^ College of Life Science and Technology Guangxi University Nanning China; ^2^ Plant Protection Research Institute, Guangxi Academy of Agricultural Science Key Laboratory of Green Prevention and Control on Fruits and Vegetables in South China Ministry of Agriculture and Rural Affairs, Guangxi Key Laboratory of Biology for Crop Diseases and Insect Pests Nanning China

**Keywords:** ATPase activity, DrdR, PilB/PilT, Tandem receiver, *Xanthomonas campestris* pv. *campestris* (Xcc)

## Abstract

Like many other bacterial organisms, *Xanthomonas campestris* pv. *campestris* (*Xcc*) employs two motility appendages, a single polar flagellum and type IV pili, for sensing environmental cues and traversing liquids and solid surfaces. However, compared to the flagellum, the molecular mechanisms underlying the function and regulation of the pilus in *Xcc* and other xanthomonads are not fully understood. In this study, we demonstrated that a special conserved orphan response regulator DrdR, which contains double receiver (REC) domains but lacks an output domain, modulates the activities of motor proteins of pili and regulates bacterial motilities, chemotaxis, and virulence in *Xcc*. Deletion of *drdR* in *Xcc* enhanced the flagellum‐dependent swimming motility, inhibited pilus‐dependent swarming motility, and altered swimming‐chemotaxis. In addition, loss of DrdR led to reduced virulence and hydathode colonization. Protein–protein interaction and enzymatic assays demonstrated that DrdR physically interacts with the pili motor proteins PilB and PilT, enhancing their ATPase activity. Moreover, *in silico* analysis combined with site‐directed mutagenesis revealed that the C‐terminal REC domain is a functional receiver, whereas the N‐terminal REC domain is a catalytically inactive pseudo‐receiver. Taken together, our findings suggest that *Xcc* DrdR most likely controls pilus‐dependent swarming motility through modulating the ATPase activities of two key motor proteins, PilT and PilB, and this motility appears to be vital for the early invasion of *Xcc*.

## Impact statement

Like many bacteria, *Xanthomonas campestris* pv. *campestris* (*Xcc*) uses a polar flagellum and type IV pili to navigate through liquids and over solid surfaces, both of which are essential for its adaptation to dynamic circumstances. Compared to the flagellum, knowledge of pilus function and regulation in *Xcc* and other motile bacteria remains limited. Here, we report that a unique response regulator (RR), DrdR, a member of the group of RRs containing tandem receiver domains that are widespread in bacteria, controls pilus‐dependent swarming motility through modulating the ATPase activities of two key motor proteins, PilT and PilB. To our knowledge, this study provides an evidence that a tandem receiver domain RR mediates its regulatory effect through directly interacting with target proteins.

## INTRODUCTION

Most prokaryotic bacteria have evolved motility machinery and employ various mechanisms to navigate through liquids and over solid surfaces^1^. This ability enables them to move toward favorable environments or escape from unfavorable ones and effectively compete with other microorganisms. The surface appendage flagella are the most common and well‐studied motility devices. Many motile bacteria use flagella for swimming through liquid environments, while in some species, flagella also enable swarming across moist surfaces^1^.

Type IV pili (T4P) are another type of motile surface appendage found in many Gram‐negative bacteria and certain Gram‐positive bacteria. Beyond mediating motilities on solid surfaces, such as twitching, swarming, and sliding, T4P are also involved in several other important physiological processes in some bacteria, including biofilm formation and surface sensing^1^, ^2^, ^3^. Furthermore, T4P are often considered more important than flagella in mediating interactions with host surfaces.

Functions and biogenesis of T4P have been explored in several animal‐pathogenic bacteria, such as *Pseudomonas aeruginosa* and *Myxococcus xanthus* ^1^, ^2^, ^3^. T4P are thin, flexible filaments that are primarily composed of thousands of PilA (the major pilin) subunits, along with minor pilins such as FimU and FimT. The filaments can be swiftly extended and retracted through the assembly and disassembly of PilA monomers into polymers, respectively, pulling bacterial cells over a moist surface to which the filaments are attached. The extension is powered by PilB, a hexameric ATPase and motor protein located on the bacterial inner membrane, whereas the retraction relies on another cytoplasmic hexameric ATPase, PilT, as well as an accessory protein PilU^2^, ^4^, ^5^, ^6^, ^7^.

While many motile bacteria possess either flagella or T4P for movement, *Xanthomonas campestris* pv. *campestris* (*Xcc*), a pathogen responsible for black rot disease in cruciferous plants, employs both types of motile appendages: a single polar flagellum for swimming and T4P for swarming^8^, ^9^, ^10^, a coordinated form of movement that allows bacterial communities to rapidly translocate over a surface. As a member of the genus *Xanthomonas*, a group of Gram‐negative bacteria that includes diverse plant pathogens infecting approximately 400 plant hosts, *Xcc* serves as a model for studying plant–microbe interactions^11^. Motility is considered necessary for *Xcc* and many other *Xanthomonas* species and pathovars to attach to host surfaces and cause disease. However, only a few studies have elucidated the functions and regulatory mechanisms of motility in xanthomonads. To date, how the flagellum and T4P coordinate to regulate cell motility remains unclear.

Several single‐domain response regulators (SD‐RRs), which consist of a receiver (REC) domain only, of two‐component signaling systems (TCSs) have been demonstrated to regulate cell motility via direct interaction with flagellar or pilus component proteins, such as the binding of *Escherichia coli* CheY to the flagellar proteins FliM and FliN^12^, the interaction of *P. aeruginosa* PilG and PilH with the pilus proteins PilB and PilT^13^, ^14^, and the binding of *X. campestris* VemR to FliM^15^. Genome sequence analysis revealed the presence of a unique RR, designated DrdR (double receiver domain RR), which is composed of two REC domains but lacks any output domain, in most *Xanthomonas* species and pathovars. Further examination of genomic data indicated that tandem receiver‐containing RRs, as well as histidine kinases (HKs) containing such tandem domains, are widespread among bacteria (https://www.genome.jp/kegg). Here, we show that *Xcc* DrdR, encoded by the *XC_3845* gene in strain 8004, is involved in virulence, swimming and swarming motilities, and chemotaxis. We provide evidence that DrdR regulates swarming motility by modulating the ATPase activity of the pilus motor proteins PilB and PilT in *Xcc* and that this motility is required during the early stage of host invasion.

## RESULTS

### DrdR has divergent effects on swimming and swarming motilities and plays a role in chemotaxis and early invasion

The *Xcc* DrdR protein is 280 amino acids in length and consists of two tandem REC domains, which we designated as REC1 (amino acid residues 14–129) and REC2 (amino acid residues 141–264), respectively (Figure S1A). It is considered an orphan RR, as no histidine kinase (HK) gene is found in proximity to the *drdR* gene in the *Xcc* genome. A sequence analysis revealed that DrdR is highly conserved within the *Xanthomonas* genus, with most sequenced *Xanthomonas* spp. exhibiting more than 90% amino acid sequence identity with the *Xcc* DrdR (Figure S1B). In order to explore the precise role of DrdR in *Xcc*, a mutant strain designated Δ*drdR* was generated (Table S1). Additionally, the complemented strain CΔ*drdR* (Table S1) was generated.

**Figure 1:**
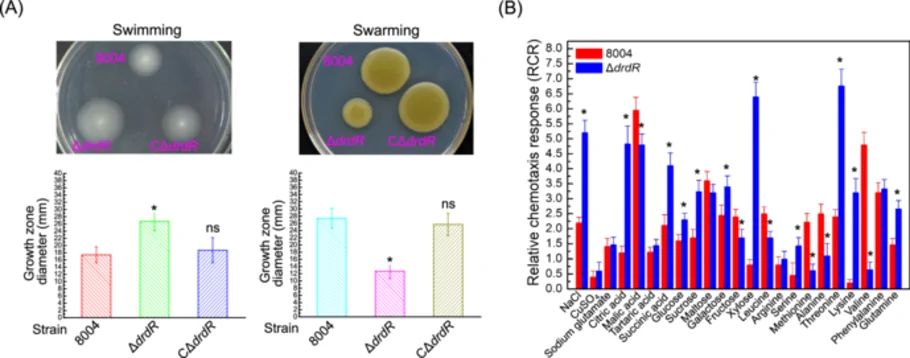
Deletion of DrdR in *Xanthomonas campestris* pv. *campestris* (*Xcc*) affects bacterial motility and chemotaxis. (A) DrdR has a divergent effect on swimming and swarming motilities. *Xcc* strains were stabbed into “swim” plates or spotted onto “swarm” plates, followed by incubation for 4 and 3 days, respectively. The representative colony morphologies of *Xcc* strains were photographed (up), and colony diameters of each strain on different media were measured (down). Statistical significance was determined by one‐way analysis of variance (ANOVA) followed by Dunnett's post hoc test. Values given are the mean ± SD (*n* = 10 colonies). *p < 0.05. ns, not significant. Similar results were obtained in two other independent experiments. (B) The *drdR* deletion mutant exhibits altered chemotaxis responses to different compounds. The relative chemotaxis response (RCR) was calculated as the ratio of bacterial cells in test capillaries to those in control capillaries. Values given are the mean ± SD (*n* = 5). The asterisk on each column indicates significantly different values compared to the values of the wild‐type 8004 strains (Student's *t* test; *p < 0.05).

*Xcc* exhibits swimming and swarming motilities, which are mediated by its polar flagellum and T4P, respectively^8^, ^16^. To determine whether DrdR affects swimming motility, *Xcc* strains (including wild‐type strain 8004, Δ*drdR*, and CΔ*drdR*) were stabbed into semi‐solid agar plates (0.28% agar). The result revealed that the diameters of Δ*drdR* colonies on swimming media increased by 53.5% relative to the wild‐type strain (Figure 1A), indicating an enhancement of swimming motility. In contrast, CΔ*drdR* restored the swimming ability of the Δ*drdR* mutant to the wild‐type level (Figure 1A). To investigate if DrdR is involved in pilus‐dependent motility, *Xcc* strains were inoculated on the surface of semi‐solid agar plates (0.6% agar). After incubating at 28°C for 3 days, the diameters of Δ*drdR* colonies were reduced by 53.7% compared with the wild‐type strain, indicating an inhibition in swarming motility. Importantly, the swarming ability of CΔ*drdR* was also restored to the wild‐type level (Figure 1A). These findings suggest that DrdR functions as a positive regulator of pilus‐based swarming but acts as a negative regulator of flagellum‐based swimming.

We further examined bacterial growth, extracellular polysaccharide (EPS) production, and the activities of several extracellular enzymes (protease, endoglucanase, and amylase), which are known to influence virulence in *Xcc* and many other pathogens of the genus *Xanthomonas*. However, no significant differences were observed between the Δ*drdR* mutant and the wild‐type strain in these phenotypic traits (Figure S2). To determine whether DrdR plays a role in bacterial chemotaxis, 23 different agents were used to compare the chemotactic responses between the wild‐type strain and the Δ*drdR* mutant. As shown in Figure 1B, compared to the wild type, the Δ*drdR* mutant exhibited significantly stronger chemotaxis toward NaCl, citric acid, succinic acid, glucose, sucrose, galactose, xylose, serine, threonine, lysine, and glutamine. Conversely, it showed significantly reduced chemotaxis toward malic acid, fructose, leucine, methionine, alanine, and valine. This suggests that DrdR differentially regulates the chemotactic response to various agents in *Xcc*.

**Figure 2:**
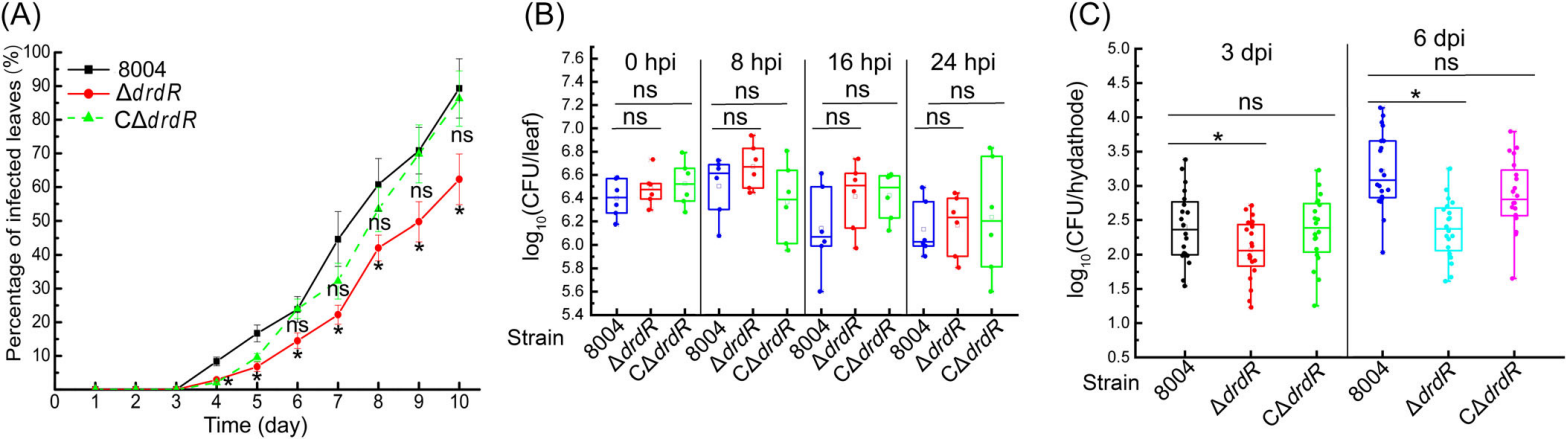
Mutation of DrdR reduces the infection of *Xcc* in host plant Chinese radish (*Raphanus sativus*). (A) Analysis of the Δ*drdR* mutant's virulence in host plants inoculated via spraying inoculation assay. The relative virulence was determined daily until 10 days post inoculation (dpi). Similar results were obtained in two other independent experiments. (B) Bacterial numbers on leaf surfaces measured at 8‐h intervals till 24 h post inoculation (hpi). Data represent the mean ± SD (*n* = 3). Similar results were obtained in two other independent experiments. (C) Quantification of the bacterial colonization in hydathodes. Each data point in the plot represents the bacterial population recovered from a single infected hydathode. A total of 20 infected hydathodes were counted for each strain. Statistical significance in (A–C) was determined by one‐way ANOVA followed by Dunnett's post hoc test. *p < 0.05; ns, not significant.

To explore the role of DrdR in the pathogenesis of *Xcc*, we first assessed the virulence of the *Xcc* strains on host plant Chinese radish (*Raphanus sativus*) using leaf‐clipping inoculation assays. However, regardless of a reduction in the mean lesion lengths, the Δ*drdR* mutant showed no reduction in virulence compared to the wild‐type strain (Figure S3). Furthermore, we evaluated the virulence of *Xcc* strains using spraying inoculation assays. The Δ*drdR* mutant displayed reduced virulence, as evidenced by a markedly lower percentage of inoculated leaves exhibiting disease symptoms compared to the wild‐type strain (Figure 2A). In contrast, the CΔ*drdR* strain restored virulence to wild‐type level (Figure 2A). Naturally, *Xcc* enters through hydathodes or wounds to cause V‐shaped necrosis on the leaves after successful establishment^17^. To determine whether the deletion of *drdR* impacts the multiplication of *Xcc* in hydathodes of the host plant, the bacterial cell numbers of *Xcc* strains in hydathodes of spraying‐inoculated leaves were estimated. Concurrently, bacterial growth on the host leaf surface was also measured at 8‐h intervals up to 24 h post‐inoculation (hpi). During the observation period, the bacterial numbers of the ∆*drdR* mutant on the leaf surfaces were similar to those of the wild‐type strain (Figure 2B). However, the ∆*drdR* mutant exhibited significantly reduced multiplication within hydathodes. Specifically, at 3 and 6 days post‐inoculation (dpi), the number of living cells of the Δ*drdR* mutant recovered from hydathodes was 47.6% and 14.9% of wild‐type levels, respectively, compared to that of the wild‐type strain (Figure 2C), while the CFU (colony‐forming unit) of the CΔ*drdR* strain was similar to that of the wild‐type strain, indicating the involvement of DrdR during hydathode infection in *Xcc*.

Overall, our findings indicate that deletion of the *drdR* gene in *Xcc* leads to a reduction in bacterial virulence and hydathode colonization on Chinese radish plants when evaluated using the leaf spraying method. This suggests that DrdR functions during the early stages of infection, likely facilitating the initial invasion of host tissues by *Xcc*.

### DrdR interacts with pili motor proteins PilB and PilT, but not PilG and PilH

**Figure 3:**
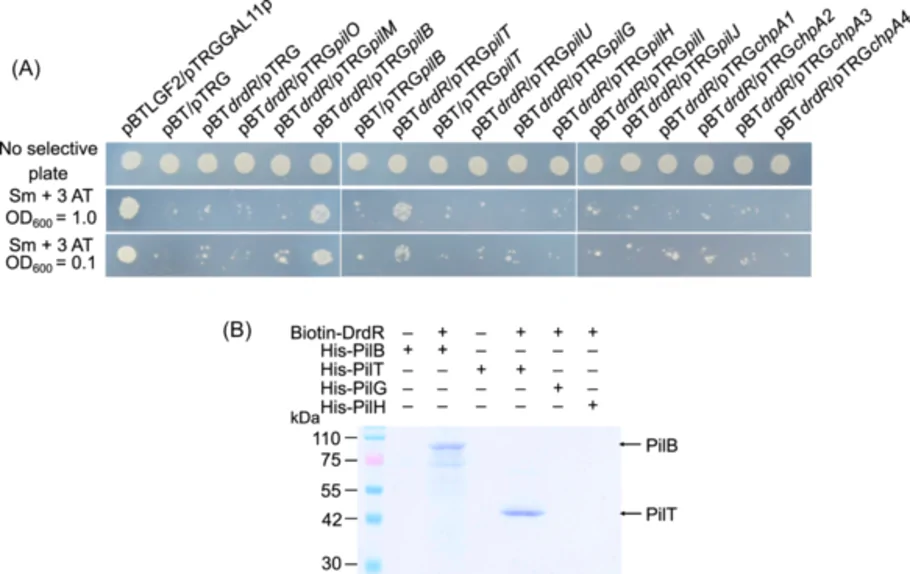
Physical interaction test between DrdR and pilus‐related proteins. (A) Bacterial two‐hybrid (B2H) assay showing interaction levels of DrdR with pilus‐related proteins. *Escherichia coli* cells with different plasmid pairs were inoculated on non‐selective plates and double‐selective indicator plates (inoculated at OD_600_ of 1.0 or 0.1). The plasmid pair pBTLGF2/pTRGGAL11p served as a positive control. Cells with plasmid pair pBT*drdR*/pTRG*pilB* or pBT*drdR*/pTRG*pilT* were able to grow on the selective plates with 3‐amino‐1,2,4‐triazole (3‐AT) and streptomycin (Sm). (B) Interactions between DrdR and PilB or PilT confirmed by *in vitro* pull‐down assay. Purified proteins (6× His‐tagged PilB, PilT, PilG, or PilH) were individually incubated with immobilized, biotinylated DrdR. After elution, samples were subjected to electrophoresis.

Given that several SD‐RRs are known to function through interactions with target proteins, we hypothesized that DrdR might similarly exert its regulatory effects via protein–protein interactions. To identify DrdR‐interacting proteins, we first performed co‐immunoprecipitation (Co‐IP) assays followed by liquid chromatography tandem‐mass spectrometry (LC‐MS/MS) analysis^18^. The reporter strain *Xcc* 8004/*drdR*::*3× flag* (Table S1) was constructed and verified (Figure S4). DrdR::3× Flag‐associated protein complexes in *Xcc* cells were immunoprecipitated and analyzed by LC‐MS/MS. By excluding background proteins from the negative control, specific DrdR‐interacting partners were identified, yielding a number of candidate proteins for further validation (Table S3). Notably, several proteins, such as PilO (XC_0939), PilM (XC_0941), PilB (XC_1060), PilT (XC_1358), PilU (XC_1359), PilG (XC_1183), and PilH (XC_1184), are involved in T4P biogenesis and regulation. To validate whether the interactions between DrdR and T4P‐associated proteins exist, bacterial two‐hybrid (B2H) assays were performed. The bait plasmid pBT*drdR* and prey plasmids (pTRG*pilO*, pTRG*pilM*, pTRG*pilB*, pTRG*pilT*, pTRG*pilU*, pTRG*pilG*, and pTRG*pilH*) were constructed. *E. coli* cells containing different plasmid pairs (Table S1) were inoculated on stringent double‐selective indicator plates. Interestingly, cells co‐expressing DrdR with either PilB or PilT exhibited colony growth. However, no growth was observed for cells co‐expressing DrdR with other T4P proteins such as PilO, PilM, PilU, PilG, or PilH, as well as those negative control cells, on the selective plates. This result demonstrated that DrdR most likely interacted with PilB and PilT (Figure 3A). To further confirm the interactions between DrdR and Pil proteins, recombinant 6× His‐tagged proteins were overexpressed in *E. coli* and purified (Figure S5), and *in vitro* protein pull‐down assays revealed that biotin‑DrdR could capture PilB and PilT (Figure 3B), but not PilG or PilH, providing direct biochemical evidence that DrdR interacts physically with PilB and PilT.

Given that DrdR controls T4P‐mediated motility, we further tested its potential interactions with several members of the Pil‐Chp system, such as PilI (XC_1185), PilJ (XC_1186), and ChpA (XC_1187), using B2H assays. However, no detectable interactions between DrdR and the Pil‐Chp proteins were observed under the test conditions (Figure 3A).

Taken together, the results from the *in vivo* Co‐IP, B2H, and *in vitro* pull‐down assays demonstrate that DrdR physically interacts with PilB and PilT. These findings suggest that DrdR may directly regulate the biogenesis and functional dynamics of T4P in *Xcc*.

### DrdR modulates the ATPase activity of the pili motor proteins PilB and PilT

**Figure 4:**
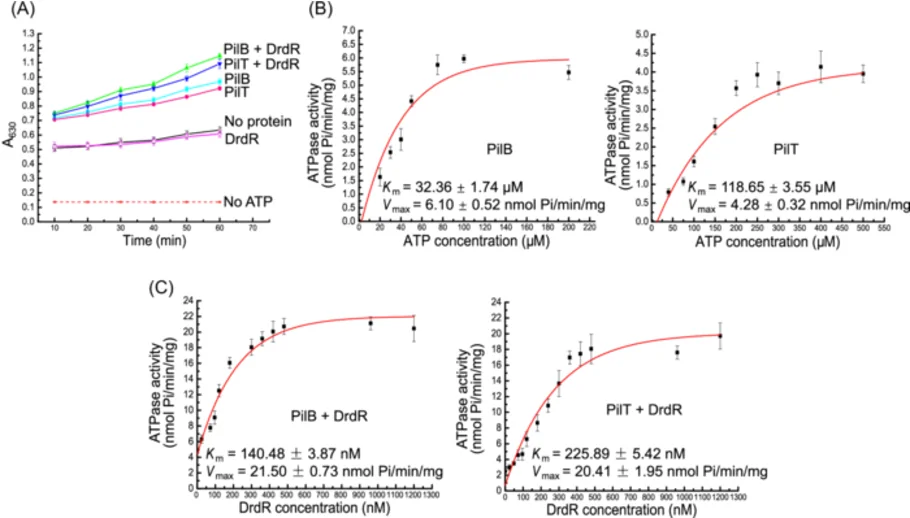
DrdR affects the ATPase activities of PilB and PilT. (A) Time‐dependent ATP hydrolysis activities of purified 6× His‐tagged proteins PilB and PilT. PilB or PilT protein and ATP were incubated at regular time intervals, followed by incubation with malachite green reagent, then the value of absorbance at 630 nm (A_630_), which reflects the release of inorganic phosphate (Pi), was measured. The addition of DrdR protein to the reaction mixture resulted in more Pi release. Each data point represents the mean ± SD from triplicate measurements. (B) Michaelis–Menten plots of PilB and PilT ATPase activity. ATP hydrolysis activity of PilB and PilT was measured across a range of ATP concentrations. Reactions that contained PilB and PilT protein and different concentrations of ATP were incubated for 30 min, and the amount of released Pi was quantified. The data shown are from a representative experiment; the points are the mean ± SD of triplicate measurements. (C) Michaelis–Menten plots of the effect of DrdR on PilB and PilT ATPase activity. Michaelis–Menten plots show the effect of DrdR on PilB and PilT ATPase activity as a function of DrdR concentration. PilB or PilT protein and ATP were incubated for 30 min at each concentration of DrdR. The data shown are from a representative experiment; Data represent the mean ± SD (*n* = 3).

The extension/assembly and retraction/disassembly of T4P are powered by the ATPase activities of PilB and PilT, respectively, as demonstrated in *M. xanthus* and *P. aeruginosa* ^4^, ^5^, ^6^, ^7^. To determine whether *Xcc* PilB and PilT have ATPase activity, we tested *in vitro* the ability of purified 6× His‐tagged proteins to hydrolyze ATP over time using the malachite green‑based method^19^. The result showed that inorganic phosphate (Pi) release amount catalyzed by *Xcc* PilB or PilT was linearly correlated with incubation time (Figure 4A), confirming the ATPase activity of *Xcc* PilB and PilT *in vitro*, which is consistent with those previously reported for their homologs in other bacterial species. Notably, this activity was enhanced upon the addition of DrdR (2 μg) (Figure 4A), indicating that DrdR stimulates the ATPase activities of these motor proteins. The ATPase activities of *Xcc* PilB and PilT, calculated by converting absorbance values to enzyme velocities using a standard curve, were further quantitatively measured as a function of ATP concentration. The Michaelis–Menten kinetic parameters (*K*_m_ and *V*_max_) were subsequently analyzed using the double‐reciprocal Lineweaver–Burk plot method. The apparent *K*_m_ values and the specific activities were 32.36 ± 1.74 μM and 6.10 ± 0.52 nmol Pi/min/mg for PilB protein and 118.65 ± 3.55 μM and 4.28 ± 0.32 nmol Pi/min/mg for PilT protein (Figure 4B), respectively. However, the activities of PilB and PilT were increased by approximately 3.5‐ and 4.8‐fold, respectively, across a range of DrdR concentrations (Figure 4C). Additionally, DrdR exhibited a higher affinity for PilB than for PilT, with corresponding *K*_m_ values of 140.48 ± 3.87 nM and 225.89 ± 5.42 nM, respectively. Taken together, these data indicate that DrdR modulates the ATPase activity of the pili motor proteins PilB and PilT.

### PilB and PilT may function downstream of DrdR in the regulatory pathway controlling swarming motility

**Figure 5:**
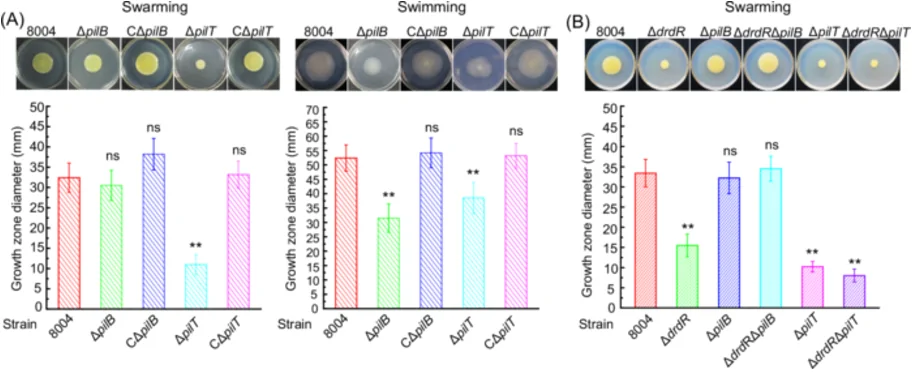
DrdR functions upstream of PilB and PilT in regulatory pathways controlling swarming motility in *Xcc*. (A) pilB and pilT involved in swarming and swimming motilities. Wild‐type 8004, Δ*pilB*, CΔ*pilB*, Δ*pilT*, and CΔ*pilT* were inoculated onto “swarming” plates or into “swimming” agar plates and incubated for 3–4 days. The representative colony morphologies of *Xcc* strains were photographed, and the colony diameters of each strain on/in plates were measured. (B) Epistasis analysis of *drdR* with *pilB* and *pilT* for swarming motility. Double‐deletion mutants of *drdR*/*pilB* and *drdR*/*pilT* exhibit similar swarming motility to that of the single deletion mutants of *pilB* and *pilT*, respectively. *Xcc* WT 8004, Δ*drdR*, Δ*pilB*, Δ*drdR*Δ*pilB*, Δ*pilT*, and Δ*drdR*Δ*pilT* strains were inoculated onto “swarming” plates and incubated for 3 days, and colony morphologies (up) and diameters (down) were documented. Statistical significance in (A) and (B) was determined by one‐way ANOVA followed by Dunnett's post hoc test. Data represent the mean ± SD (*n* = 10). **p < 0.01; ns, not significant. Similar results were obtained in two other independent experiments.

To estimate the impact of PilB and PilT activities on the motility of *Xcc*, the mutant strains Δ*pilB* and Δ*pilT* were generated (Table S1). Simultaneously, the complemented strains CΔ*pilB* and CΔ*pilT* were also constructed (Table S1). Swarming and swimming motilities of these newly constructed *Xcc* strains were tested using the corresponding agar plates. As shown in Figure 5A, the Δ*pilB* mutant exhibited similar swarming motility compared to the wild‐type strain under the tested conditions, wheras the CΔ*pilB* displayed slightly enhanced swarming with slightly larger colonies than the wild type. However, the Δ*pilT* mutant displayed almost no swarming motility with much smaller colony diameters than the wild‐type strain, and the CΔ*pilT* strain restored its motility to near wild‐type level. Interestingly, both Δ*pilB* and Δ*pilT* mutants showed a moderate reduction in swimming motility (Figure 5A), with smaller colony diameters on swimming plates compared to the wild‐type strain, and complementation restored swimming motility in both mutants. These data demonstrate that functional PilB and PilT are involved in swarming and swimming motilities in *Xcc*.

To further assess whether DrdR controls swarming motility via PilB and PilT, double‐deletion mutant Δ*drdR*Δ*pilB* and Δ*drdR*Δ*pilT* (Table S1) were created. Epistasis analysis of swarming motility revealed that the Δ*drdR*/Δ*pilT* mutant displayed a phenotype similar to the single‐deletion mutant Δ*pilT* (Figure 5B). While Δ*pilT* caused much more significant inhibition of growth than Δ*pilB*, it did not cause further defects in Δ*drdR* cells, suggesting that PilT and DrdR function in the same pathway and PilT may act downstream of DrdR to accelerate swarming motility. Interestingly, although the Δ*drdR* mutant exhibited reduced swarming motility compared to the wild type, the Δ*drdR*Δ*pilB* double mutant restored the impaired swarming motility to the wild‐type level (Figure 5B), suggesting that PilB and DrdR function in the same pathway and PilB may act downstream of DrdR to inhibit swarming motility.

Collectively, our genetic data suggest that PilB and PilT may be key components functioning downstream of, or interacting with, the DrdR regulatory network that controls swarming motility in *Xcc*. Our biochemical analysis demonstrates that DrdR directly stimulates the ATPase activities of both PilB and PilT *in vitro*. Together, these findings suggest that DrdR most likely controls swarming motility through modulating the ATPase activities of two key downstream components, PilT and PilB.

### DrdR consists of a degenerate receiver alongside a canonical receiver

Unlike the so‐called SD‐RRs, which contain only a single REC domain, DrdR comprises two tandem REC domains (Figure S1A). To determine whether individual REC domains play a regulatory role in the phenotypes controlled by DrdR, we conducted several genetic complementation assays. Results showed that neither REC1 nor REC2 coding sequence could restore the motility deficiency of the Δ*drdR* mutant (Figure S6), implying that the structural integrity of DrdR is essential for its regulatory functions.

**Figure 6:**
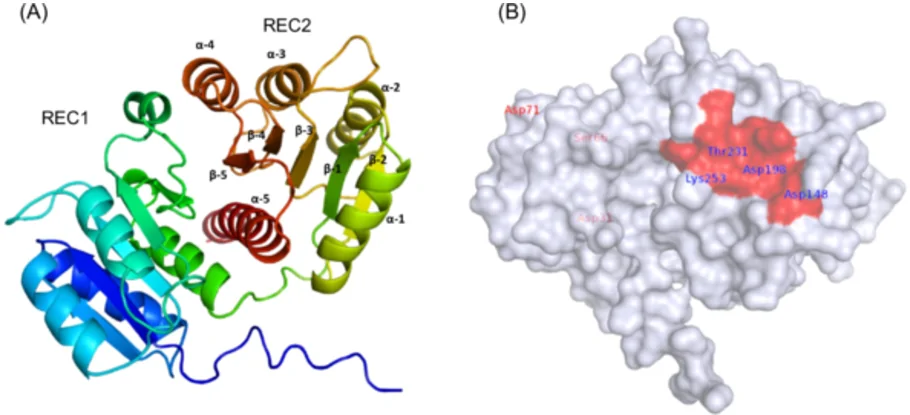
*In silico* analysis of the predicted receiver (REC) domains within DrdR. (A) Predicted structure and topology of DrdR. The structure was predicted using AlphaFold3 and rendered with PyMOL. The C‐terminal region (red to yellow) corresponds to a canonical receiver domain (REC2) with a complete (β/α)_5_ topology, characterized by five parallel β‐strands forming a central sheet flanked by five α‐helices. This arrangement forms a conserved acidic pocket at the C‐terminal end of the β‐sheet. In contrast, the N‐terminal region (blue to green) constitutes an atypical receiver domain (REC1) with a truncated (β/α)_4_ architecture, lacking the fifth β‐strand (β_5_) and its associated α_5_‐helix required for a complete active site. α‐Helices and β‐strands are shown as coiled ribbons and flat arrows, respectively. The N‐ and C‐termini are colored blue and red, respectively. (B) Predicted catalytic pocket within the REC domain. Analysis was performed using CASTpFold (https://cfold.bme.uic.edu/castpfold) on the AlphaFold3‐predicted structure. The molecular surface of the DrdR structure (white) is shown, with the putative phosphorylation pocket in the REC2 domain highlighted in red. Key pocket‐forming residues are labeled: the predicted metal‐ion binding residue Asp148, the phosphoacceptor Asp198, and the scaffold‐stabilizing residues Thr231 and Lys253. Conserved residues in the REC1 domain (e.g., Asp31, Ser66, and Asp71) are also labeled, although no analogous pocket was detected in this region.

*In silico* structural modeling of DrdR was performed using AlphaFold3. The predicted structure reveals a distinct architecture composed of two REC domains with divergent structural integrity (Figure 6A). The REC2 domain exhibits the characteristic (β/α)₅ topology of functional receiver domains, with five parallel β‐strands forming a central sheet flanked by five α‐helices, while the REC1 domain consists of only four β‐strands and four α‐helices, forming a truncated (β/α)₄ fold, suggesting that REC1 is a catalytically inactive pseudo‐receiver domain^20^. To further characterize the functional regions, potential phosphorylation pockets in DrdR were analyzed using the CASTpFold server. A prominent and well‐defined pocket (highlighted in red) was identified within the REC2 domain (Figure 6B). This pocket contains several critical residues, such as Asp148 and Asp198, predicted to serve as the metal‐ion binding residue and phosphoacceptor, respectively, and Thr231 and Lys253, which contribute to maintaining the local α‑helix–β‑strand–α‑helix scaffold essential for the active site geometry^21^. In contrast, no similar well‐defined pocket was found in the REC1 domain, despite the presence of several conserved residues (e.g., Asp31, Ser66, and Asp71) that are implicated in phosphorylation based on the literature of SD‐RR activation.

**Figure 7:**
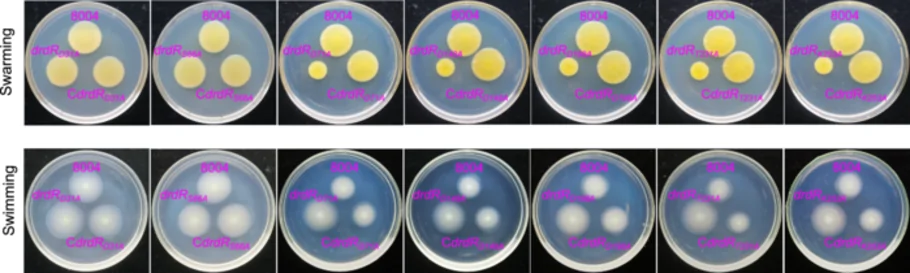
DrdR protein with replacement of Asp71, Asp148, Asp198, Thr231, or Lys253 loses its regulatory function on cell motility. Motilities of *drdR* point mutant strains *drdR*_*D31A*_, *drdR*_*S66A*_, *drdR*_*D71A*_, *drdR*_*D148A*_, *drdR*_*D198A*_, *drdR*_*T231A*_, and *drdR*_*K253A*_ were tested using “swarming” plates (up) and “swimming” plates (down). The *drdR*_*D71A*_, *drdR*_*D148A*_, *drdR*_*D198A*_, *drdR*_*T231A*_, and *drdR*_*K253A*_ mutants exhibited altered swarming and swimming motilities. For complementation, the corresponding mutants were transformed with plasmid pLCdrdR. Expression of the wild‐type copy of the *drdR* gene in these mutants restored their motilities to that seen in the wild‐type 8004 strain.

In order to evaluate the functional significance of these conserved residues (Asp31, Ser66, Asp71, Asp148, Asp198, Thr231, and Lys253) in DrdR, site‐directed mutagenesis was performed by individually replacing each residue with alanine. Complementation assays showed that introducing *drdR* alleles with substitutions at positions 71, 148, 198, 231, or 253 into the Δ*drdR* mutant failed to restore motility to wild‐type levels (Figure S7), indicating that residues Asp71, Asp148, Asp198, Thr231, and Lys253 in DrdR are essential for its regulatory function in *Xcc*. Point mutations were introduced into the chromosomal *drdR* gene, generating *Xcc* derivatives *drdR*_*D31A*_, *drdR*_*S66A*_, *drdR*_*D71A*_, *drdR*_*D148A*_, *drdR*_*D198A*_, *drdR*_*T231A*_, *and drdR*_*K253A*_ (Table S1). Motility assays revealed that five of these point‐mutated strains (*drdR*_*D71A*_, *drdR*_*D148A*_, *drdR*_*D198A*_, *drdR*_*T231A*_, *and drdR*_*K253A*_), similar to the deletion mutant strain Δ*drdR*, displayed altered cell motilities compared to the wild‐type strain (Figure 7), and the motilities of those corresponding complementary strains (C*drdR*_*D71A*_, C*drdR*_*D148A*_, C*drdR*_*D198A*_, C*drdR*_*T231A*_, and C*drdR*_*K253A*_) were restored to the wild‐type level, demonstrating that single amino acid substitutions at Asp71, Asp148, Asp198, Thr231, and Lys253 in DrdR abolish its regulatory function.

Collectively, these findings demonstrate that the functional integrity of both REC1 and REC2 domains is essential for DrdR to execute its regulatory role in *Xcc*. Specifically, the necessity of Asp71 supports the structural and functional integrity of the REC1 domain, while the indispensability of Asp148, Asp198, Thr231, and Lys253 indicates the predicted catalytic function of the REC2 domain.

## DISCUSSION

The biogenesis and function of T4P in *Xanthomonas* species are thought to be orchestrated through an intricate network of protein interactions between the core motor proteins PilB and PilT/PilU and the accessory proteins whose activities are dynamically modulated by intracellular or extracellular signals at the pilus base^10^, for example, two c‐di‐GMP receptor proteins PilZ and FimX form a complex to control T4P biogenesis through interaction with PilB^7^. However, only a few interacting proteins of PilB or PilT/PilU have been characterized. In this study, it was found that deletion of *drdR* inhibited cell swarming motility on semi‐solid surfaces (0.6% agar) (Figure 1A), indicating that DrdR positively regulates pilus‐mediated motility in *Xcc*. *In vivo* Co‐IP and B2H and *in vitro* pull‐down assays demonstrated that DrdR interacted with both PilB and PilT (Table S3 and Figure 3). Enzymatic assays further revealed that the PilB and PilT proteins of the T4P system from *Xcc* exhibited ATPase activity *in vitro* (Figure 4), and this activity was significantly enhanced by DrdR. While the effects of DrdR‐PilB/PilT interactions on *in vivo* ATPase activity, the mechanisms by which ATP hydrolysis drives T4P extension/retraction, and the regulation of DrdR itself remain unclear, here we infer that DrdR functions as an activator that most likely controls T4P‐mediated motility through direct interaction with the motor proteins PilT and PilB in *Xcc*. To our knowledge, this work presents an experimental evidence that a tandem‐receiver RR exerts its regulatory function through direct physical interactions with its target proteins.

Swarming motility is a rapid, multicellular form of bacterial surface translocation that is driven by flagella rotation, either by T4P‐mediated pulling or slime secretion‐mediated pushing forces^1^, ^22^. In some organisms, such as *P. aeruginosa* strain PAO1, both flagella and T4P are involved in swarming^23^. In *Xcc*, several studies suggest that swarming is primarily mediated by T4P^8^, ^9^, ^24^. Similarly, our data also demonstrated that a mutation in T4P gene *pilT* abolished swarming motility (Figure 5A). Furthermore, recent studies demonstrated that deletions of flagellar gene *fliM* (*XC_2267*), *fliN* (*XC_2268*), or *cheY* (*XC_2282*) in *Xcc* abolished swimming motility, but almost had no effects on swarming motility^15^, ^24^. Our data, combined with previous findings, suggest that the swarming motility in *Xcc* requires functional T4P instead of a flagellum.

T4P have been demonstrated to exhibit divergent functions in motilities across different *Xanthomonas* spp. For instance, while twitching motility, which is mediated by the extension and retraction of T4P, was not observed in *Xcc* ^25^, it has been described in several other xanthomonads, such as *Xanthomonas citri* subsp. *citri*, *Xanthomonas oryzae* pv. *oryzae*, and *Xanthomonas oryzae* pv. *oryzicola*. In these organisms, mutants lacking a functional gene of T4P generally displayed reduced twitching motility but enhanced spreading^25^, ^26^, ^27^, ^28^. In contrast, swimming motility has long been considered independent of T4P. In this study, *Xcc* strains with a deletion of *pilB* or *pilT* exhibited attenuated swimming capacity (Figure 5A). These observations suggest that T4P in *Xcc*, unlike in many other xanthomondas, may not only mediate swarming motility but also influence swimming motility. Alternatively, these T4P‐related genes in *Xcc*, besides their roles in T4P biogenesis, might influence swimming through mechanisms that are not yet understood. Thus, PilB may regulate swimming by modulating T4P assembly or/and via T4P‐independent pathways. For example, PilB has been reported to regulate EPS production and biofilm formation in *M. xanthus* ^29^, ^30^.

Interestingly, although the PilB and PilT function downstream of DrdR in swarming, their deletion mutants exhibited divergent swimming motility phenotypes. The Δ*drdR* mutant showed enhanced swimming (Figures 1A and 5A), whereas the *pilB*/*pilT* mutants exhibited reduced swimming. This divergence suggests that DrdR likely regulates swimming motility via a pathway independent of PilB and PilT. How DrdR regulates swimming motility remains unclear and worthy of future investigation. Furthermore, the contrasting phenotypes resulting from *drdR* deletion, enhanced swimming motility but reduced swarming motility, indicate that DrdR might have different functional effects on the flagellar and T4P systems. Whether DrdR also affects the structural consequences of these motile appendages remains unknown. As electron microscopy is the gold standard for definitive morphological assessment, visualizing the flagella and pili in the Δ*drdR* mutant by this method, as well as in the *pilB*/*pilT* mutants, will be performed in our future research.

**Figure 8:**
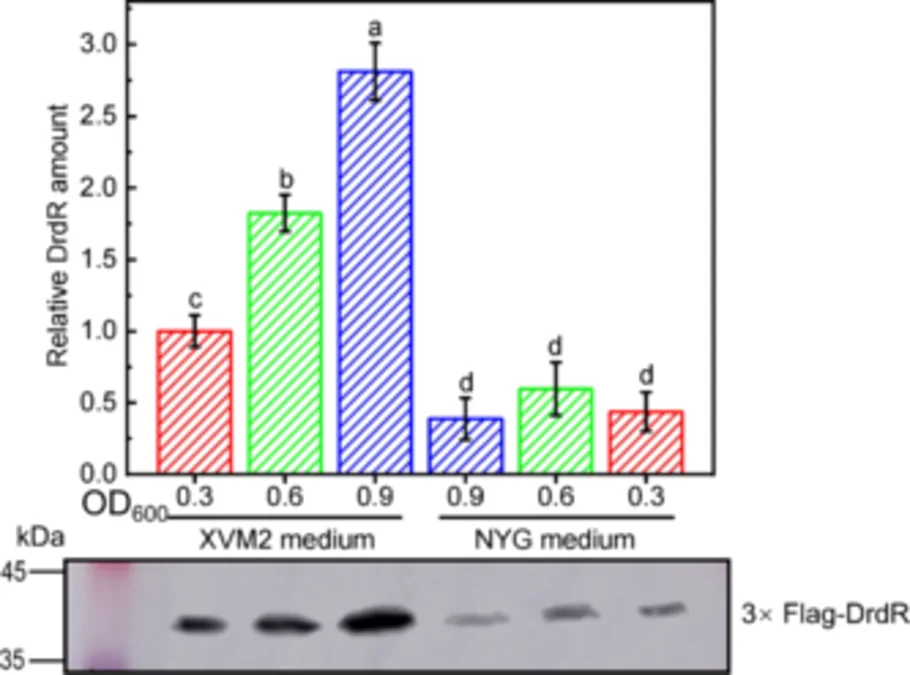
DrdR expression is induced in *Xcc* under minimal medium condition. *Xcc* wild‐type cells expressing 3× Flag‐tagged DrdR were cultured in nutrition‐rich NYG medium and minimal medium XVM2 designed to mimic the plant environment. Bacterial cells equivalent to an OD_600_ of 1.0 (volumetrically) were harvested at OD_600_ of 0.3, 0.6, and 0.9, which corresponded to the early‐, mid‐, and late‐exponential growth phases in XVM2, respectively. Total proteins were extracted from these samples and subjected to SDS‐PAGE. The 3× Flag‐tagged DrdR protein was detected by western blotting. Band intensities were quantified using ImageJ. Data are presented as means ± SD (*n* = 3). The mean value of DrdR levels in XVM2‐cultured cells at OD_600_ 0.3 was normalized to 1.0. Different lowercase letters above the columns indicate significant differences (*p* < 0.05, Student's *t*‐test). The experiment was repeated three times.

Although motility and chemotaxis have been shown to be key virulence traits, especially for many bacterial plant pathogens^31^, only limited studies have focused on these activities in *Xanthomonas* spp. Previous data suggest that motility and chemotaxis are required at the initial stages of invasion, primarily for the entry of xanthomonads into host plants^18^, ^32^. However, recent transcriptomic analysis revealed that the expression of many genes involved in the biogenesis of flagellum, pilus, and chemotaxis was strongly repressed after *Xcc* cells entered the hydathodes of host plants, suggesting that *Xcc* does not use flagellum‐dependent and pilus‐dependent motilities or chemotaxis after entering the host hydathodes^33^. Generally, *Xcc* cells grow on leaf surfaces and infiltrate the host plant via hydathodes and wounds, subsequently spreading through the leaf xylem vessels. However, the precise mechanisms by which epiphytic *Xcc* cells move to and enter host openings remain unclear. In this study, the Δ*drdR* mutant exhibited reduced multiplication in hydathodes at 3 and 6 dpi compared to the wild type following leaf spraying inoculation (Figure 2C). Additionally, the expression levels of DrdR in *Xcc* were significantly elevated in cells cultured in the minimal medium XVM2, a plant‐mimicking medium^34^, relative to the nutrition‐rich medium NY (Figure 8), and DrdR levels increased progressively during bacterial growth in XVM2. These western blotting data suggest that DrdR expression, similar to the Type III secretion system, a key virulence factor, is induced in minimal medium and likely activated during bacterial growth on plant surfaces. This finding further supports the suggestion that motility, particularly pilus‐dependent motility, and/or chemotaxis play a role in *Xcc* entry into the hydathodes.

Two‐component signaling systems (TCSs) are widely utilized by bacteria to perceive and respond to environmental changes^21^. A canonical TCS comprises a HK sensor that detects a specific signal and a cognate RR that modulates cellular processes by controlling the expression of target genes or the activity of target proteins. Upon sensing a stimulus, the HK undergoes autophosphorylation at a conserved histidine residue. This phosphoryl group is subsequently transfered to a conserved aspartate residue located within the REC domain of the RR, thereby activating the RR output domain. Recent experimental evidence has shown that some RRs can exert their regulatory functions in a non‐phosphorylated state, and many orphan RRs can be activated by alternative phosphodonors (e.g., non‐cognate HKs, serine/threonine kinases, or acetyl phosphate)^35^, ^36^. Here, genomic analysis of *Xcc* suggests DrdR is an orphan RR and *in silico* and mutational analysis indicate that the predicted catalytic pocket in REC2 is functionally essential (Figures 7 and S7). Whether DrdR requires phosphorylation to perform its function and which signal or phosphodonor activates DrdR remain unclear. The detailed mechanism through which DrdR regulates swarming motility, as well as swimming motility, is worthy of future investigation.

Notably, although RRs (and even some HKs) containing tandem REC domains are widespread in bacteria, the functional understanding of such dual‐receiver systems remains limited. Our *in silico* analysis suggests that the N‐terminal REC1 domain of DrdR is catalytically inactive (Figure 6), whereas mutational studies indicate that this degenerate REC domain contains at least one indispensable residue (Asp71) (Figures 7 and S7). This finding implies that REC1 plays a vital role in DrdR regulatory function. This domain, or its conserved residues, may contribute to structural stabilization, intramolecular signal propagation, or even serve as an output domain mediating protein–protein interactions. Although the detailed mechanism of REC1 requires further investigation, our model, in which DrdR operates through an integrated mechanism requiring both a canonical catalytic receiver and a non‐catalytic yet functionally essential pseudo‐receiver, sheds new light on the functional mechanisms of those tandem‐receiver TCS proteins.

## MATERIALS AND METHODS

### Bacterial strains, plasmids, culture media, and growth conditions

The strains and plasmids employed in this work are detailed in Table S1. *Xcc* strains were cultivated in nutrient‐yeast‐glycerol (NYG) medium (per liter: 5 g peptone, 3 g yeast extract, and 20 g glycerol) or in NY medium (NYG without glycerol) at 28°C. *E. coli* strains were incubated at 37°C in Luria–Bertani (LB) medium (per liter: 5 g yeast extract, 10 g NaCl, and 10 g tryptone) or on LB agar plates (LB supplemented with 15 g agar per liter). Antibiotics were used as described in our previous studies^15^, ^19^.

### Gene deletion and functional complementation analysis

Deletion strain of *drdR*, *pilB*, or *pilT* was generated by allelic homologous recombination using the pK18mob*sacB*‐mediated method as our previously described^15^, ^19^. Briefly, 200–500 bp DNA fragments corresponding to the upstream and downstream regions flanking the target gene coding sequence were amplified by PCR from genomic DNA of *Xcc* strain 8004 with primers specified in Table S2. The two fragments were subsequently cloned in tandem into the pK18mob*sacB* vector (Table S1). The resulting recombinant plasmids (pKΔ*drdR*, pKΔ*pilB*, and pKΔ*pilT*; see Table S1) were transferred into the *Xcc* strain 8004 via triparental mating. Putative mutants were isolated on appropriate selective agar plates and confirmed by DNA sequencing. The unmarked in‐frame deletion mutants obtained were designated Δ*drdR*, Δ*pilB*, and Δ*pilT* (Table S1), respectively.

For complementation, the coding sequences of *drdR*, *pilB*, and *pilT* were amplified by PCR from *Xcc* strain 8004 using gene‐specific primer pairs (Table S2). Each amplified fragment was individually cloned into the broad‐host‐range vector pLAFR3 (Table S1), generating the recombinant plasmids pLC*drdR*, pLC*pilB*, and pLC*pilT* (Table S1). These plasmids were then transferred into their respective gene deletion mutants via mating, resulting in complemented strains designated CΔ*drdR*, CΔ*pilB*, and CΔ*pilT* (Table S1).

To generate the double‐deletion mutants of *drdR*/*pilB* and *drdR*/*pilT*, the previously constructed recombinant plasmids pKΔ*pilB* and pKΔ*pilT* (Table S1) were separately introduced into the *drdR* single‐deletion mutant Δ*drdR*, and transconjugants were selected on appropriate selective plates. The resulting double‐deletion mutants were named Δ*drdR*Δ*pilB* and Δ*drdR*Δ*pilT* (Table S1), respectively.

### Site‐directed mutagenesis

Site‐directed mutagenesis was performed using the Mut Express II Fast Mutagenesis Kit V2 (Vazyme). Briefly, the *drdR* gene was first cloned into the vector pUC19, generating the recombinant plasmid pU*drdR* (Table S1). Mutagenic PCR was then performed using pU*drdR* as the template with the oligonucleotide primers listed in Table S2. The resulting PCR products were treated with *Dpn*I and subsequently transformed into the *E. coli* strain DH5α, yielding recombinant plasmids containing *drdR* with specific codon mutations. The mutated *drdR* genes were subcloned into the pLAFR3 vector, generating recombinant plasmids pLC*drdR*_D31A_, pLC*drdR*_S66A_, pLC*drdR*_D71A_, pLC*drdR*_D148A_, pLC*drdR*_D198A_, pLC*drdR*_T231A_, and pLC*drdR*_K253A_ (Table S1). These plasmids, which contain *drdR* alleles carrying alanine substitutions at residues Asp31, Ser66, Asp71, Asp148, Asp198, Thr231, and Lys253, were used for subsequent functional complementation assays.

To generate *Xcc drdR* mutants with single amino acid substitutions, the mutated *drdR* alleles were individually subcloned into the pK18mob*sacB* vector (Table S1), generating recombinant plasmids pKB*drdR*_*D31A*_, pKB*drdR*_*S66A*_, pKB*drdR*_*D71A*_, pKB*drdR*_*D148A*_, pKB*drdR*_*D198A*_, pKB*drdR*_*T231A*_, and pKB*drdR*_*K253A*_ (Table S1). These plasmids were separately introduced into the *Xcc* wild‐type strain 8004 via triparental mating. Putative point mutant derivatives were isolated and confirmed. The DrdR point mutants obtained were designated *drdR*_*D31A*_, *drdR*_*S66A*_, *drdR*_*D71A*_, *drdR*_*D148A*_, *drdR*_*D198A*_, *drdR*_*T231A*_, and *drdR*_*K253A*_ (Table S1), respectively.

### B2H assay

To assess the potential interactions between DrdR and its candidate target proteins, the BacterioMatch II system (Stratagene) was employed as previously described^15^. Briefly, the coding sequences of the *drdR* gene and the candidate genes (*pilO*, *pilM*, *pilB*, *pilT*, *pilU*, *pilG*, *pilH*, *pilI*, *pilJ*, *chpA1‐4*, *cheA*, and *cheY*) were individually PCR‐amplified using the genomic DNA of *Xcc* strain 8004 as the template, with the corresponding primer sets listed in Table S2. These fragments were subsequently cloned into the pBT (bait) or pTRG (prey) vectors, generating the bait plasmid pBT*drdR* and the prey plasmids pTRG*pilO*, pTRG*pilM*, pTRG*pilB*, pTRG*pilT*, pTRG*pilU*, pTRG*pilG*, pTRG*pilH*, pTRG*pilI*, pTRG*pilJ*, pTRG*chpA1‐4*, pTRG*cheA*, and pTRG*cheY* (Table S1). *E. coli* XL1‐Blue MRF′ cells, co‐transformed with different bait and prey plasmid pairs, were grown on the nonselective plates and double‐selective indicator plates contain 3‐amino‐1,2,4‐triazole (3‐AT) and streptomycin (Sm).

### Overexpression and purification of proteins

To overproduce 6× His‐tagged forms of *Xcc* DrdR, PilB, PilT, PilG, and PilH, the coding sequences of *drdR* (840 bp), *pilB* (1731 bp), *pilT* (1035 bp), *pilG* (399 bp), and *pilH* (360 bp) were PCR‐amplified using the corresponding primer sets (Table S2) and cloned into the expression vector pET‐30a or pET‐32a. The resulting recombinant plasmids (Table S1) were individually transformed into *E. coli* strain BL21 (DE3). Transformed strains (Table S1) were grown in LB medium and induced with isopropyl β‐d‐thiogalactopyranoside (IPTG). Cells were harvested, and the 6× His‐tagged proteins were purified using Ni‐NTA affinity resin (Qiagen).

### Protein pull‐down assay

To examine protein–protein interactions *in vitro*, the ProFound pull‐down biotinylated protein–protein interaction kit (Pierce) was employed as previously described^15^. Briefly, 50 μl of purified 6× His‑tagged DrdR protein (0.5 mg/ml), biotinylated with sulfo‐NHS‐LC‐biotin, was incubated with streptavidin sepharose beads. After thorough washing, the beads were incubated individually with 6× His‑tagged PilB, PilT, PilG, or PilH. After additional washes, the bound prey proteins were eluted. Eluates were run on 12% SDS‐PAGE gel and stained with Coomassie blue.

### Co‐IP and LC‐MS/MS analysis

Co‐IP and LC–MS/MS analysis were performed to identify the potential DrdR‐interacting partners, as previously described^18^ with minor modifications. To construct an *Xcc* strain chromosomally encoded 3× Flag‐tagged DrdR protein (*drdR*::3× *flag*), two DNA fragments, one consisting of 519 bp of DNA upstream of the *drdR* stop codon and a 41‐bp flag‐coding sequence and the other consisting of 40 bp flag‐coding sequence, 3‐bp stop codon, and 542 bp downstream of the *drdR* stop codon, were generated by PCR amplification using *Xcc* 8004 genomic DNA as the template, with the corresponding primer sets (Table S2). These fragments were joined together by overlap extension PCR and cloned into pK18mob*sacB* (Table S1) to generate pK*drdR*::*flag* (Table S1), which was then conjugated into *Xcc* strain 8004. The variant strain was selected and confirmed. The obtained strain was named 8004/*drdR*::*3× flag* (Table S1).

*Xcc* variant strain 8004/*drdR*::*3× flag* and wild‐type strain 8004 (control) were grown in the NYG medium, and total proteins were prepared. For each sample, 50 μl of agarose‐conjugated anti‐Flag was added and incubated. After washing the agarose beads according to standard protocols, the bound proteins were eluted. The eluted proteins were analyzed by LC‐MS/MS using a Nano‐LC system coupled with an LTQ‐Orbitrap Elite mass spectrometer. The MS data were analyzed using SEQUEST against the *Xcc* UniProt protein database. Peptides were filtered with a confidence level of 99%, and the false discovery rate (FDR) for peptide identification was set to 1%.

### Western blotting

Western blotting was performed as previously described^19^. Briefly, proteins were separated by SDS‐PAGE and transferred to a PVDF membrane (Millipore). After blocking, the membrane was incubated with the primary anti‐Flag mouse monoclonal antibody (1:2500; Solarbio), washed with Tris‐buffered saline with Tween buffer, and then probed with the second antibody horseradish peroxidase‐conjugated (HRP) goat anti‐mouse IgG (1:2500). Signals were detected by chemiluminescence.

### ATPase assay

The ATPase activities of purified 6× His‐tagged PilB and PilT were analyzed using the malachite green phosphate detection kit (Beyotime) according to the procedures described in our previous study^19^. In brief, reactions contained 2 μg of either PilB or PilT protein, in either the absence or presence of 2 μg DrdR protein. Reactions were performed in reaction buffer containing 1 mM ATP (or varying ATP concentrations for kinetic assays) and incubated at 30°C for 30 min (or at regular time intervals). An aliquot from each reaction was added to a 96‐well microplate, followed by the addition of freshly prepared phosphate assay reagent. After incubation at 30°C for 30 min, the optical density was measured at 630 nm. Reactions without any protein, or ATP, or with DrdR only served as negative controls. The amount of Pi released was quantified using a standard curve.

### Cell motility assay

The motilities of *Xanthomonas* strains were assessed as described in our previous studies^15^, ^19^. In brief, for swimming motility, bacterial suspensions (OD_600_ of 1.0) were stabbed into 0.28% agar plates (containing Bacto peptone and yeast extract) and incubated for 3–4 days. For swarming motility, suspensions were inoculated on the surfaces of 0.6% agar NA plates (containing 2% glucose), followed by incubation for 3 days. The diameter of the area colonized by each strain was measured.

### Chemotaxis assay

Chemotaxis assays were conducted as previously described^18^. Briefly, 100 μl of *Xcc* cultures (approximately 10^9^ CFU/ml) were aspirated into a pipette tip. A capillary tube filled with chemoattractant solution was securely positioned at the tip of pipette containing the bacterial suspension for 2 h. Then the contents of the capillary were serially diluted to approximately 10^5^ CFU/ml and plated onto an NYG plate. Bacterial colonies were counted after 3 days of incubation. A parallel control assay was conducted using a capillary containing PBS instead of chemoattractant. The relative chemotaxis response was expressed as the ratio of CFU recovered from the test capillary to that from the control capillary.

### Virulence assay in plant

The virulence of *Xcc* to the host plant Chinese radish (*Raphanus sativus*) was evaluated using a spraying method. Cells from bacterial cultures were harvested, washed, and resuspended in sterile ultrapure water to a density of OD_600_ = 0.01 (approximately 10^7^ CFU/ml). Approximately 200 leaves were sprayed with 50 ml of the bacterial suspension per treatment. Three replicates were performed for each independent experiment. Bacterial virulence was calculated as the percentage of inoculated leaves displaying disease symptoms at the leaf margins.

Bacterial populations in hydathodes were quantified at 3 and 6 dpi^33^. Hydathodes were sampled using a 1.5 mm diameter punch (five leaves per *Xcc* strain, one leaf per plant, at least five hydathode per leaf) and homogenized. The homogenates were serially diluted in NYG medium, and 100 μl of each dilution was plated onto agar plates for colony counting. Bacterial populations were calculated as log_10_CFU per hydathode after 3 days of incubation. Bacterial growth on leaf surfaces was measured every 8 h post‐inoculation until 24 h. For each strain, two leaves per plant from five plants were sampled per treatment. Each leaf sample was homogenized in 9 ml of sterile water.

### Statistical analyses

Statistical significance was determined by one‐way analysis of variance (ANOVA) followed by Dunnett's post hoc test, unless otherwise specified. Data are presented as means ± standard deviation (SD) of measurements (or treatments) in a representative experiment. All experiments were performed at least three times independently.

## AUTHOR CONTRIBUTIONS

**Peidong Ren**: Data curation; formal analysis; investigation; methodology; writing—original draft; writing—review and editing. **Jianrou Meng**: Investigation; methodology; validation. **Qijian Qin**: Formal analysis; investigation; validation. **Jiafeng Shi**: Investigation; visualization. **Jiali Yao**: Data curation; investigation. **Yanhua Qi**: Investigation. **Jiliang Tang**: Formal analysis; project administration; resources; supervision. **Rui‐Fang Li**: Conceptualization; methodology; project administration; supervision; validation; writing—original draft; writing—review and editing. **Guang‐Tao Lu**: Conceptualization; data curation; formal analysis; funding acquisition; methodology; project administration; supervision; writing—original draft; writing—review and editing.

## ETHICS STATEMENT

This study did not involve human subjects or animals.

## CONFLICT OF INTERESTS

The authors declare no conflict of interests.

## Supporting information

Supporting Information file 1.

Table S1. Bacterial strains and plasmids used in this work.

Table S2. Primers used in this work.

Table S3. The potential target proteins of DrdR identified by Co‐IP coupled with LC‐MS/MS assays.

## Data Availability

The data that support the findings of this study are available from the corresponding authors upon reasonable request.
