# Supplementary material for: Tandem receiver domain response regulator DrdR modulates swarming by controlling PilB/PilT activity in Xanthomonas campestris
Source: mLife. 2026 Aug 25;5(4):473–85. doi: 10.1002/mlf2.70092 (PMC13507683; doi:10.1002/mlf2.70092)
Supplement: Supplementary file 1 [file MLF2-5-473-s001.doc]

**Table S3.** The potential target proteins of DrdR identified by Co-IP coupled with LC-MS/MS assays

| **Gene ID** | **Name** | **Description** |
| --- | --- | --- |
| *XC_0449* |  | Transcriptional regulator MarR/EmrR family |
| *XC_0638* | *mcp* | Methyl-accepting chemotaxis protein |
| *XC_0728* | *sreK* | Two-component system sensor protein |
| *XC_0730* | *sreS* | Two-component system sensor protein |
| *XC_0752* |  | LacI family transcriptional regulator |
| *XC_0830* |  | TetR/AcrR family transcriptional regulator |
| *XC_0850* |  | Response regulator |
| *XC_0938* | *pilP* | Fimbrial assembly protein |
| *XC_0939* | *pilO* | Fimbrial assembly membrane protein |
| *XC_0941* | *pilM* | Fimbrial assembly membrane protein |
| *XC_0984* |  | Transcriptional regulator LysR family |
| *XC_1049* | *colR* | Two-component system regulatory protein |
| *XC_1050* | *colS* | Two-component system sensor protein |
| *XC_1056* | *pilD* | Type IV pre-pilin leader peptidase |
| *XC_1057* | *pilC* | Fimbrial assembly protein |
| *XC_1058* | *pilA* | Type IV pilus assembly protein |
| *XC_1059* | *pilA* | Type IV pilus assembly protein |
| *XC_1060* | *pilB* | Type IV pilus assembly protein |
| *XC_1186* | *PilJ* | Twitching motility protein |
| *XC_1187* | *pilL* | Themosensory pili system protein ChpA |
| *XC_1240* | *tfmR* | TetR famliy transcriptional regulator |
| *XC_1358* | *pilT* | Twitching motility protein |
| *XC_1359* | *pilU* | Twitching motility protein |
| *XC_1621* | *fimT* | Type IV fimbrial biogenesis protein |
| *XC_1626* | *pilE* | Type IV pilus assembly protein |
| *XC_1691* | *fixL* | Sensor histidine kinase |
| *XC_1824* | *fimX* | Multidomain signaling protein |
| *XC_1925* | *ihfB* | Integration host factor subunit beta |
| *XC_2201* | *pilF* | Fimbrial biogenesis protein |
| *XC_2227* | *ravS* | Two-component system sensor protein |
| *XC_2228* | *ravR* | Two-component system regulatory protein |
| *XC_2229* | *ravA* | Two-component system sensor protein |
| *XC_2250* |  | Response regulator |
| *XC_2252* | *vemR* | Response regulator |
| *XC_2268* | *fliN* | Flagellar motor switch protein |
| *XC_2284* | *cheA* | Two-component system sensor protein |
| *XC_2311* | *mcp* | Methyl-accepting chemotaxis protein |
| *XC_2320* | *mcp* | Methyl-accepting chemotaxis protein |
| *XC_2506* | *rsmA* | Carbon storage regulator |
| *XC_2639* |  | Transcriptional regulator lysR family |
| *XC_2767* | *fur* | Fur family transcriptional regulator |
| *XC_2801* | *sstF* | Transcriptional regulator lysR family |
| *XC_3057* |  | Two-component system sensor protein |
| *XC_3117* |  | Two-component system regulatory protein |
| *XC_3221* | *pilZ* | Type IV pilus assembly protein |
| *XC_3428* |  | GntR family transcriptional regulator |
| *XC_3451* | *colS/detS* | Two-component system sensor protein |
| *XC_3597* | *xrvB* | DNA-binding protein H-NS |
| *XC_3823* | *pilA* | Fimbrial protein |
| *XC_3985* |  | Histone H1 |
