# Supplementary material for: Tandem receiver domain response regulator DrdR modulates swarming by controlling PilB/PilT activity in Xanthomonas campestris
Source: mLife. 2026 Aug 25;5(4):473–85. doi: 10.1002/mlf2.70092 (PMC13507683; doi:10.1002/mlf2.70092)
Supplement: Supplementary file 2 [file MLF2-5-473-s004.doc]

**Table S2 Primers used in this study^§^**

| **Primer** | **Nucleotide sequence (5′→3′)** | **The amplified fragment or the utilization** |
| --- | --- | --- |
| L*drdR*-FlagF  L*drdR*-FlagR | CGGGATCCTTCACCGAATACGTCACCGA  ATGTCATGATCTTTATAATCACCGTCATGGTCTTTGTAGTCTAGCGTGACGGCCTGATC | 560-bp DNA fragment containing 519-bp DNA fragmen of *drdR* (*XC_3845*) ORF sequence and 41-bp Flag-coding sequence. Used for constructing *Xcc* strain producing DrdR::3×Flag protein. |
| R*drdR*-FlagF  R*drdR*-FlagR | TAAAGATCATGACATCGACTACAAGGATGACGATGACAAGTGACCGAAGGCGAGGGGC  CCCAAGCTTATCGATCACTTTGCCCTTGG | 585-bp DNA fragment containing 40-bp Flag-coding sequence, 3-bp stop codon and 542-bp downstream of the *drdR* stop codon. Used for constructing *Xcc* strain producing DrdR::3×Flag protein. |
| L*drdR*-F  L*drdR*-R | ACAGTTGGATCCCAAGCGGCTAGATGGCTGGCTGCAT  ACAGTTTCTAGACCCATCGACCACCATCACACGCGG | 528-bp DNA sequence upstream of *Xcc* *drdR* (*XC_3845*), used for constructing the *drdR* deletion mutant. |
| R*drdR*-F  R*drdR*-R | ACAGTTTCTAGA TGACCGAAGGCGAGGGGCGTATCC  ACAGTTAAGCTT CCGTCAGCACCGCATTGAGCAGCA | 423-bp DNA sequence downstream of *Xcc* *drdR* (*XC_3845*), used for constructing the *drdR* deletion mutant. |
| *CdrdR-*F  *CdrdR-*R | CGGGATCCATGCATCCACCAGATCTCAA  CCCAAGCTTTCATAGCGTGACGGCCTGAT | 840-bp DNA fragment of *Xcc* (or *Xoc*, or *Xoo*) *drdR* coding sequence. Clone into the vector pLAFR3 for complementation, or expression vector pET-32a for overexpression. |
| REC1*-*F  REC1*-*R | CGGGATCCATGCATCCACCAGATCTCAA  CCCAAGCTTTCAGATGAAGGTCGCCAGCG | 387-bp DNA coding sequence of *drdR* REC1 domain with 3-bp stop codon. |
| REC2*-*F  REC2*-*R | CGGGATCCATGACCGTGCTGTACATCGA  CCCAAGCTTTCACACCTTGGTCACCAGCA | 369-bp DNA coding sequence of *drdR* REC2 domain with 3-bp start and stop codon. |
| D31-F  D31-R | ATTGCCGcCGTGCTCAAGCGCGACTTGCCCAA  TTGAGCACGgCGGCAATCAGCTTGCGTACCAG | Used for *Xcc* *drdR* site-directed mutagenesis, replacing Asp-31 to Ala-31. |
| S66-F  S66-R | ACCACCgCGTTGTCGTTACCCGATGGCGACGG  AACGACAACGcGGTGGTGACCAGATCCACCGC | Used for *Xcc* *drdR* site-directed mutagenesis, replacing Ser-66 to Ala-66. |
| D71-F  D71-R | TTGTCGTTACCCGcTGGCGACGGCCTGACGCT  CCAgCGGGTAACGACAACGAGGTGGTGACCAG | Used for *Xcc drdR* site-directed mutagenesis, replacing Asp71 to Ala. |
| D148-F  D148-R | TGTACATCGAGGcCAGCCGCGTGGTGGCCGAA  GCTGgCCTCGATGTACAGCACGGTGGCGCCCA | Used for *Xcc* *drdR* site-directed mutagenesis, replacing Asp148 to Ala. |
| D198-F  D198-R | ACCGcCGTCACCCTCAAGGGCGAGCTCAACGG  TTGAGGGTGACGgCGGTCAGCACCACGTCGAT | Used for *Xcc* *drdR* site-directed mutagenesis, replacing Asp198 to Ala. |
| T231-F  T231-R | ATGgCCGGCGACACCAACCCGCGCAACCAGTC  TTGGTGTCGCCGGcCATCACCAGCACCGGCAG | Used for *Xcc drdR* site-directed mutagenesis, replacing Thr231 to Ala. |
| K253-F  K253-R | CAAgcGCCCATCGAAGAACGCCTGCTGGTGACC  TTCTTCGATGGGCgcTTGCACCAGGTCGTTGGC | Used for *Xcc* *drdR* site-directed mutagenesis, replacing Lys253 to Ala. |
| L*pilB*-F  L*pilB*-R | CGGGATCCTTGATCAGAAGTATCTGC  GCTCTAGAACGCTCATTACGCACCTA | 636-bp DNA sequence upstream of *pilB*, used for constructing the pilB deletion mutant. |
| R*pilB*-F  R*pilB*-R | GCTCTAGAACCAAGGACTGACAGGCCG  CCCAAGCTTATCGATCCGGCCTCCTCT | 598-bp DNA sequence downstream of *pilB*, used for constructing the *pilB* deletion mutant. |
| L*pilT*-F  L*pilT*-R | CGGGATCCAGCAGTGCGATCAGCTTG  GCTCTAGAGCGATATCCATGCGCTGC | 609-bp DNA sequence upstream of *pilT*, used for constructing the *pilT* deletion mutant. |
| R*pilT*-F  R*pilT*-R | GCTCTAGA AAGCGGATATTCGAGTGACT  CCCAAGCTTAATTCTGGTTGCGGTAGC | 600-bp DNA sequence downstream of *pilT*, used for constructing the *pilT* deletion mutant. |
| *pilB*-OF  *pilB*-OR | CGGAATTCATGAGCGTTGTGCTAACGG  CCCTCGAGTCAGTCCTTGGTCACACG | 1731-bp DNA fragment of the *pilB* gene coding sequence. Used for overexpression and pull-down assays. |
| *pilT*-OF  *pilT*-OR | CCCAAGCTTCGATGGATATCGCTGAAC  CCCTCGAGTCACTCGAATATCCGCTT | 1035-bp DNA fragment of the *pilT* gene coding sequence. Used for overexpression and pull-down assays. |
| *pilG-*OF  *pilG-*OR | CGGGATCCATGACTGAAAACATGGCT  CCCTCGAGTCAGGCGTTGACGTACGT | 399-bp DNA fragment of the *pilG* gene coding sequence. Used for overexpression and pull-down assays. |
| *pilH-*OF  *pilH-*OR | CGGGATCCATGGCTCGAATCATATTG  CCCTCGAGTCAACGCACCAGTTGTTT | 360-bp DNA fragment of the *pilH* gene coding sequence. Used for overexpression and pull-down assays. |
| *drdR*-BTF  *drdR*-BTR | CGGAATTCCATGCATCCACCAGATCTC  CGGGATCCTCATAGCGTGACGGCCTG | 840-bp DNA fragment of the *drdR* gene coding sequence. Cloned into the bait vector pBT for bacterial two-hybrid assays. |
| *pilB*-TRGF  *pilB*-TRGR | CGGAATTCAGATGAGCGTTGTGCTAACG  CCCTCGAGTCAGTCCTTGGTCACACG | 1731-bp DNA fragment of the *pilB* gene coding sequence. Cloned into target vector pTRG for bacterial two-hybrid assays. |
| *pilT*-TRGF  *pilT*-TRGR | CGGAATTCAGATGGATATCGCTGAAC  GGACTAGTTCACTCGAATATCCGCTT | 1035-bp DNA fragment of the *pilT* gene coding sequence. Cloned into target vector pTRG for bacterial two-hybrid assays. |
| *pilO*-TRGF  *pilO*-TRGR | CGGGATCCATGAGTAAGAAATCATTCAA  CCCTCGAGTCATTGGCCGCCCTTCTTCG | 663-bp DNA fragment of the *pilO* gene coding sequence. Cloned into target vector pTRG for bacterial two-hybrid assays. |
| *pilM*-TRGF  *pilM*-TRGR | CGGGATCCGTGCCCGTCGGCAGGGGA  CCCTCGAGTCAGTCAAAGCTCCTCAG | 1080-bp DNA fragment of the *pilM* gene coding sequence. Cloned into target vector pTRG for bacterial two-hybrid assays. |
| *pilU*-TRGF  *pilU*-TRGR | CGGGATCC ATGAGCACCATCGACTTC  CCCTCGAGTTACCGAACCTCGGCAAT | 1128-bp DNA fragment of the *pilU* gene coding sequence. Cloned into target vector pTRG for bacterial two-hybrid assays. |
| *pilG*-TRGF  *pilG*-TRGR | CGGGATCCATGACTGAAAACATGGCT  CCCTCGAGTCAGGCGTTGACGTACGT | 399-bp DNA fragment of the *pilG* gene coding sequence. Cloned into target vector pTRG for bacterial two-hybrid assays. |
| *pilH*-TRGF  *pilH*-TRGR | CGGGATCCATGGCTCGAATCATATTG  CCCTCGAGTCAACGCACCAGTTGTTT | 360-bp DNA fragment of the *pilH* gene coding sequence. Cloned into target vector pTRG for bacterial two-hybrid assays. |
| *pilI*-TRGF  *pilI*-TRGR | CGGGATCCTTGATGCGTTCTCCATTCGA  CCCTCGAGTTAGGCCGCGGCCTGCCGGA | 531-bp DNA fragment of the *pilI* gene coding sequence. Cloned into target vector pTRG for bacterial two-hybrid assays. |
| *pilJ*-TRGF  *pilJ*-TRGR | CGGGATCCCGCGAGCAGGACGTGCGCT  CCCTCGAGTCACGCCGGCAGCTTGAA | 1035-bp DNA fragment of the *pilJ* gene coding sequence. Cloned into target vector pTRG for bacterial two-hybrid assays. |
| *chpA1*-TRGF  *chpA1*-TRGR | CGGGATCCATGGGCCAAGCGCCGCAATT  CCCTCGAGCAGCAGTTCTTCCTTGAC | 600-bp DNA fragment of part of the *chpA* gene coding region. Cloned into target vector pTRG for bacterial two-hybrid assays. |
| *chpA2*-TRGF  *chpA2*-TRGR | CGGGATCCAACCCGTTCGTGGAATCG  CCCTCGAGTTCGTCGACTGCAATTGG | 1353-bp DNA fragment of part of the *chpA* gene coding region. Cloned into target vector pTRG for bacterial two-hybrid assays. |
| *chpA3*-TRGF  *chpA3*-TRGR | CGGGATCCGCTGCGCAGTTGCGCGAC  CCCTCGAGCAGGCCGCCCAAGTCGGC | 1053-bp DNA fragment of part of the *chpA* gene coding region. Cloned into target vector pTRG for bacterial two-hybrid assays. |
| *chpA4*-TRGF  *chpA4*-TRGR | CGGGATCCGGCAAGCAGGTGCATCTG  CCCTCGAGTTATTCTCGGACACGGGC | 1323-bp DNA fragment of part of the *chpA* gene coding region. Cloned into target vector pTRG for bacterial two-hybrid assays. |
| *cheA*-TRGF  *cheA*-TRGR | CGGGATCCATGAGTGCTGTTCCAGAC  CCCTCGAGTCAATGATCGCTGGAACG | 1653-bp DNA fragment of the *cheA* gene coding sequence. Cloned into target vector pTRG for bacterial two-hybrid assays. |

^§^The underlined sequences indicate the restriction sites for *Bam*HI, *Eco*RI, *Hin*dIII, *Kpn*I, *Pst*I, *Sac*I, *Xba*I and *Xho*I, respectively. The long square boxes indicate the Flag-coding sequences, and highlights in gray show the complementary sequences. Lowercase letters indicate the substitution of nucleotides.
