# Supplementary material for: Tandem receiver domain response regulator DrdR modulates swarming by controlling PilB/PilT activity in Xanthomonas campestris
Source: mLife. 2026 Aug 25;5(4):473–85. doi: 10.1002/mlf2.70092 (PMC13507683; doi:10.1002/mlf2.70092)
Supplement: Supplementary file 3 [file MLF2-5-473-s002.doc]

**Table S1. Bacterial strains and plasmids used in this study**

| **Strains or plasmids** | **Relevant characteristics** | **Reference or source** |
| --- | --- | --- |
| ***Escherichia coli* strains** |  |  |
| DH5α | Φ80△*lacZM*15 *recA1 endA1 deoR* | Gibco BRL, Life Technologies |
| BL21(DE3) | F^-^ *ompT gal dcm lon hsdS_B_* (*r^-^_B_ m^-^_B_*) λ(DE3) | Novagen, Germany |
| BL21/ pET-30a -PilT | BL21(DE3) harboring plasmid pET-30a -PilT | This work |
| BL21/pET-30a -PilG | BL21(DE3) harboring plasmid pET-30a -PilG | This work |
| BL21/pET-30a -PilH | BL21(DE3) harboring plasmid pET-30a -PilH | This work |
| BL21/pET-32a-DrdR | BL21(DE3) harboring plasmid pET-32a-DrdR | This work |
| BL21/pET-32a -PilB | BL21(DE3) harboring plasmid pET-32a -PilB | This work |
| XL1-Blue MRF' | Reporter strain, Δ*(mcrA)183* Δ*(mcrCB-hsdSMR-mrr)173 endA1 hisB supE44 thi-1 recA1 gyrA96relA1 lac* [F*´ lacIq HIS3 aadA* Kan*^r^*^]^ | Stratagene |
| XL1-Blue MRF'/pBT*drdR*/pTRG | XL1-Blue MRF' harboring plasmids pBT*drdR* and pTRG | This work |
| XL1-Blue MRF'/pBT*drdR*/pTRG*pilO* | XL1-Blue MRF' harboring plasmids pBT*drdR* and pTRG*pilO* | This work |
| XL1-Blue MRF'/pBT*drdR*/pTRG*pilM* | XL1-Blue MRF' harboring plasmids pBT*drdR* and pTRG*pilM* | This work |
| XL1-Blue MRF'/pBT*drdR*/pTRG*pilB* | XL1-Blue MRF' harboring plasmids pBT*drdR* and pTRG*pilB* | This work |
| XL1-Blue MRF'/pBT*drdR*/pTRG*pilT* | XL1-Blue MRF' harboring plasmids pBT*drdR* and pTRG*pilT* | This work |
| XL1-Blue MRF'/pBT*drdR*/pTRG*pilU* | XL1-Blue MRF' harboring plasmids pBT*drdR* and pTRG*pilU* | This work |
| XL1-Blue MRF'/pBT*drdR*/pTRG*pilG* | XL1-Blue MRF' harboring plasmids pBT*drdR* and pTRG*pilG* | This work |
| XL1-Blue MRF'/pBT*drdR*/pTRG*pilH* | XL1-Blue MRF' harboring plasmids pBT*drdR* and pTRG*pilH* | This work |
| XL1-Blue MRF'/pBT*drdR*/pTRG*pilI* | XL1-Blue MRF' harboring plasmids pBT*drdR* and pTRG*pilI* | This work |
| XL1-Blue MRF'/pBT*drdR*/pTRG*pilJ* | XL1-Blue MRF' harboring plasmids pBT*drdR* and pTRG*pilJ* | This work |
| XL1-Blue MRF'/pBT*drdR*/pTRG*chpA1* | XL1-Blue MRF' harboring plasmids pBT*drdR* and pTRG*chpA1* | This work |
| XL1-Blue MRF'/pBT*drdR*/pTRG*chpA2* | XL1-Blue MRF' harboring plasmids pBT*drdR* and pTRG*chpA2* | This work |
| XL1-Blue MRF'/pBT*drdR*/pTRG*chpA3* | XL1-Blue MRF' harboring plasmids pBT*drdR* and pTRG*chpA3* | This work |
| XL1-Blue MRF'/pBT*drdR*/pTRG*chpA4* | XL1-Blue MRF' harboring plasmids pBT*drdR* and pTRG*chpA4* | This work |
| XL1-Blue MRF'/pBT*drdR*/pTRG*cheA* | XL1-Blue MRF' harboring plasmids pBT*drdR* and pTRG*cheA* | This work |
| XL1-Blue MRF'/pBT/pTRG*pilB* | XL1-Blue MRF' harboring plasmids pBT and pTRG*pilB* | This work |
| XL1-Blue MRF'/pBT/pTRG*pilT* | XL1-Blue MRF' harboring plasmids pBT and pTRG*pilT* | This work |
| ***Xanthomonas campestris* pv. *campestris* strains** |  |  |
| 8004 | Wild-type strain. Rif^r^ | Daniels et al., 1984 |
| Δ*drdR* | As 8004, but *XC_3845* (*drdR*) gene deleted, non-polar effect. Rif^r^ | This work |
| CΔ*drdR* | Δ*drdR* harboring a recombinant plasmid pLC*drdR* derived from the ORF of *drdR* gene cloned into the plasmid pLAFR3. Rif^r^ Tet^r^ | This work |
| Δ*drdR*/pLC*drdR_D31A_* | Δ*drdR* harboring a recombinant plasmid pLC*drdR_D31A_* derived from the point-mutated *drdR* cloned into the plasmid pLAFR3. Rif^r^ Tet^r^ | This work |
| Δ*drdR*/pLC*drdR_S66A_* | Δ*drdR* harboring a recombinant plasmid pLC*drdR_S66A_* derived from the point-mutated *drdR* cloned into the plasmid pLAFR3. Rif^r^ Tet^r^ | This work |
| Δ*drdR*/pLC*drdR*_D71A_ | Δ*drdR* harboring a recombinant plasmid pLC*drdR*_D71A_ derived from the point-mutated *drdR* cloned into the plasmid pLAFR3. Rif^r^ Tet^r^ | This work |
| Δ*drdR*/pLC*drdR*_D148A_ | Δ*drdR* harboring a recombinant plasmid pLC*drdR*_D148A_ derived from the point-mutated *drdR* cloned into the plasmid pLAFR3. Rif^r^ Tet^r^ | This work |
| Δ*drdR*/pLC*drdR*_D198A_ | Δ*drdR* harboring a recombinant plasmid pLC*drdR*_D198A_ derived from the point-mutated *drdR* cloned into the plasmid pLAFR3. Rif^r^ Tet^r^ | This work |
| Δ*drdR*/pLC*drdR*_T231A_ | Δ*drdR* harboring a recombinant plasmid pLC*drdR*_T231A_ derived from the point-mutated *drdR* cloned into the plasmid pLAFR3. Rif^r^ Tet^r^ | This work |
| Δ*drdR*/pLC*drdR*_K253A_ | Δ*drdR* harboring a recombinant plasmid pLC*drdR*_K253A_ derived from the point-mutated *drdR* cloned into the plasmid pLAFR3. Rif^r^ Tet^r^ | This work |
| Δ*pilB* | As 8004, but *XC_1060* (*pilB*) gene deleted, non-polar effect. Rif^r^ | This work |
| CΔ*pilB* | Δ*pilB* harboring a recombinant plasmid pLC*pilB* derived from the ORF of *pilB* cloned into the plasmid pLAFR3. Rif^r^ Tet^r^ | This work |
| Δ*pilT* | As 8004, but *XC_1358* (*pilT*) gene deleted, non-polar effect. Rif^r^ | This work |
| CΔ*pilT* | Δ*pilT* harboring a recombinant plasmid pLC*pilT* derived from the ORF of *pilT* cloned into the plasmid pLAFR3. Rif^r^ Tet^r^ | This work |
| Δ*drdR*Δ*pilB* | As Δ*drdR*, but *XC_1060* (*pilB*) gene deleted, non-polar effect. Rif^r^ | This work |
| Δ*drdR*Δ*pilT* | As Δ*drdR*, but *XC_1058* (*pilT*) gene deleted, non-polar effect. Rif^r^ | This work |
| 8004/*drdR::3×flag* | As 8004, but chromosomally encoding a 3×Flag fused DrdR protein. Rif^r^ | This work |
| *drdR_D31A_* | Strain 8004 derivative with *drdR* replaced *in situ* by *drdR_D31A_* | This work |
| C*drdR_D31A_* | Complemented strain, *drdR_D31A_* harboring a recombinant plasmid pLC*drdR*. Rif^r^ Tet^r^ |  |
| *drdR_S66A_* | Strain 8004 derivative with *drdR* replaced *in situ* by *drdR_S66A_* | This work |
| C*drdR_S66A_* | Complemented strain, *drdR_S66A_* harboring a recombinant plasmid pLC*drdR*. Rif^r^ Tet^r^ |  |
| *drdR_D71A_* | Strain 8004 derivative with *drdR* replaced *in situ* by *drdR_D71A_* | This work |
| C*drdR_D71A_* | Complemented strain, *drdR_D71A_* harboring a recombinant plasmid pLC*drdR*. Rif^r^ Tet^r^ |  |
| *drdR_D148A_* | Strain 8004 derivative with *drdR* replaced *in situ* by *drdR_D148A_* | This work |
| C*drdR_D148A_* | Complemented strain, *drdR_D148A_* harboring a recombinant plasmid pLC*drdR*. Rif^r^ Tet^r^ |  |
| *drdR_D198A_* | Strain 8004 derivative with *drdR* replaced *in situ* by *drdR_D198A_* | This work |
| C*drdR_D198A_* | Complemented strain, *drdR_D198A_* harboring a recombinant plasmid pLC*drdR*. Rif^r^ Tet^r^ |  |
| *drdR_T231A_* | Strain 8004 derivative with *drdR* replaced *in situ* by *drdR_T231A_* | This work |
| C*drdR_T231A_* | Complemented strain, *drdR_T231A_* harboring a recombinant plasmid pLC*drdR*. Rif^r^ Tet^r^ |  |
| *drdR_K253A_* | Strain 8004 derivative with *drdR* replaced *in situ* by *drdR_K253A_* | This work |
| C*drdR_K253A_* | Complemented strain, *drdR_K253A_* harboring a recombinant plasmid pLC*drdR*. Rif^r^ Tet^r^ | This work |
| **Plasmids** |  |  |
| pRK2073 | Helper plasmid, Tra+, Mob+, ColE1, Spc^r^. | Leong et al., 1982 |
| pUC19 | ColE1 Amp^r^ cloning vector (GenBank accession no. L09137.2) | Yanisch-Perron et al., 1985 |
| pU*drdR* | 840-bp DNA fragment of *drdR* gene coding sequence of *Xcc* strain cloned into the plasmid pUC19. Amp^r^ | This work |
| pLAFR3 | Broad host range cloning vector, Tet^r^ | Staskawicz et al., 1987 |
| pLC*drdR* | 840-bp DNA fragment of *drdR* gene coding sequence (*XC_3845*) of *Xcc* strain cloned into the plasmid pLAFR3. Tet^r^ | This work |
| pK18*mobsacB* | pUC18 derivative, *lacZα*, *sacB*, Kan^r^, *mob* site. Allelic exchange vector (Suicidal vector carrying *sacB* gene for mutagenesis) | Schäfer et al., 1994 |
| pKΔ*drdR* | pK18*mobsacB* containing *Xcc* fragments flanking *drdR*. Kan^r^ | This work |
| pKΔ*pilB* | pK18*mobsacB* containing *Xcc* fragments flanking *pilB*. Kan^r^ | This work |
| pKΔ*pilT* | pK18*mobsacB* containing *Xcc* fragments flanking *pilT*. Kan^r^ | This work |
| pK*drdR*::flag | pK18*mobsacB* containing fragment composing 519-bp DrdR-coding sequence, 66-bp 3×Flag-coding sequence, 3-bp stop codon and 542-bp downstream of the *drdR*. | This work |
| pLC*drdR_D31A_* | 840-bp DNA fragment of *Xcc* *drdR* coding sequence in which the Asp31 was replaced by Ala cloned into the plasmid pLAFR3. Tet^r^ | This work |
| pLC*drdR_S66A_* | 840-bp DNA fragment of *Xcc* *drdR* coding sequence in which the Ser66 was replaced by Ala cloned into the plasmid pLAFR3. Tet^r^ | This work |
| pLC*drdR_D71A_* | 840-bp DNA fragment of *Xcc* *drdR* coding sequence in which the Asp71 was replaced by Ala cloned into the plasmid pLAFR3. Tet^r^ | This work |
| pLC*drdR_D148A_* | 840-bp DNA fragment of *Xcc* *drdR* coding sequence in which the Asp148 was replaced by Ala cloned into the plasmid pLAFR3. Tet^r^ | This work |
| pLC*drdR_D198A_* | 840-bp DNA fragment of *Xcc* *drdR* coding sequence in which the Asp198 was replaced by Ala cloned into the plasmid pLAFR3. Tet^r^ | This work |
| pLC*drdR_T231A_* | 840-bp DNA fragment of *Xcc* *drdR* coding sequence in which the Thr231 was replaced by Ala cloned into the plasmid pLAFR3. Tet^r^ | This work |
| pLC*drdR_K253A_* | 840-bp DNA fragment of *Xcc* *drdR* coding sequence in which the Lys253 was replaced by Ala cloned into the plasmid pLAFR3. Tet^r^ | This work |
| pKB*drdR_D31A_* | 840-bp DNA fragment of *Xcc* *drdR* coding sequence in which the Asp31 was replaced by Ala cloned into the suicide plasmid pK18mob*sacB*. Kan^r^ | This work |
| pKB*drdR_S66A_* | 840-bp DNA fragment of *Xcc* *drdR* coding sequence in which the Ser66 was replaced by Ala cloned into the suicide plasmid pK18mob*sacB*. Kan^r^ | This work |
| pKB*drdR_D71A_* | 840-bp DNA fragment of *Xcc* *drdR* coding sequence in which the Asp71 was replaced by Ala cloned into the suicide plasmid pK18mob*sacB*. Kan^r^ | This work |
| pKB*drdR_D148A_* | 840-bp DNA fragment of *Xcc* *drdR* coding sequence in which the Asp148 was replaced by Ala cloned into the suicide plasmid pK18mob*sacB*. Kan^r^ | This work |
| pKB*drdR_D198A_* | 840-bp DNA fragment of *Xcc* *drdR* coding sequence in which the Asp198 was replaced by Ala cloned into the suicide plasmid pK18mob*sacB*. Kan^r^ | This work |
| pKB*drdR_T231A_* | 840-bp DNA fragment of *Xcc* *drdR* coding sequence in which the Thr231 was replaced by Ala cloned into the suicide plasmid pK18mob*sacB*. Kan^r^ | This work |
| pKB*drdR_K253A_* | 840-bp DNA fragment of *Xcc* *drdR* coding sequence in which the Lys253 was replaced by Ala cloned into the suicide plasmid pK18mob*sacB*. Kan^r^ | This work |
| pET-30a | Expression vector, allow the production of fusion proteins containing amino terminal 6×His-tagged sequences. Kanr | Novagen |
| pET-30a-PilT | pET-30a containing a 1035-bp fragment of *pilT* (*XC_1358*) gene coding sequence | This work |
| pET-30a-PilG | pET-30a containing a 399-bp fragment of *pilG* (*XC_1183*) gene coding sequence | This work |
| pET-30a-PilH | pET-30a containing a 360-bp fragment of *pilH* (*XC_1184*) gene coding sequence | This work |
| pET-32a | Expression vector, allow the production of fusion proteins containing amino terminal thioredoxin-tagged and carboxyl-terminal 6×His-tagged sequences. Amp^r^ | Novagen |
| pET-32a-DrdR | pET-32a containing an 840-bp fragment of *drdR* gene coding sequence | This work |
| pET-32a-PilB | pET-32a containing an 1731-bp fragment of *pilB* (*XC_1060*) gene coding sequence | This work |
| pBT | Two-hybrid system bait plasmid containing the *cat* gene, p15A origin of replication and λ cI ORF. | Stratagene |
| pBT*drdR* | pBT derivative carrying the full length of the coding sequence of *drdR* gene. Cat^r^ | This work |
| pTRG | Two-hybrid system target plasmid containing the *tet* gene, ColE1 origin of replication, and RNA polymerase α subunit ORF. | Stratagene |
| pTRG*pilB* | pTRG derivative carrying the coding sequence of *pilB* gene (1731-bp). Tet^r^ | This work |
| pTRG*pilT* | pTRG derivative carrying the coding sequence of *pilT* gene (1035-bp). Tet^r^ | This work |
| pTRG*pilO* | pTRG derivative carrying the coding sequence of *pilO* gene (663-bp). Tet^r^ | This work |
| pTRG*pilM* | pTRG derivative carrying the coding sequence of *pilM* gene (1080-bp). Tet^r^ | This work |
| pTRG*pilU* | pTRG derivative carrying the coding sequence of *pilU* gene (1128-bp). Tet^r^ | This work |
| pTRG*pilG* | pTRG derivative carrying the coding sequence of *pilG* gene (399-bp). Tet^r^ | This work |
| pTRG*pilH* | pTRG derivative carrying the coding sequence of *pilH* gene (360-bp). Tet^r^ | This work |
| pTRG*pilI* | pTRG derivative carrying the coding sequence of *pilI* gene (531-bp). Tet^r^ | This work |
| pTRG*pilJ* | pTRG derivative carrying the coding sequence of *pilJ* gene (1035-bp). Tet^r^ | This work |
| pTRG*chpA1* | pTRG derivative carrying a 600-bp DNA fragment of part of *chpA* gene coding region. Tet^r^ | This work |
| pTRG*chpA2* | pTRG derivative carrying a 1353-bp DNA fragment of part of *chpA* gene coding region. Tet^r^ | This work |
| pTRG*chpA3* | pTRG derivative carrying a 1053-bp DNA fragment of part of *chpA* gene coding region. Tet^r^ | This work |
| pTRG*chpA4* | pTRG derivative carrying a 1323-bp DNA fragment of part of *chpA* gene coding region. Tet^r^ | This work |
| pTRG*cheA* | pTRG derivative carrying 1653-bp coding sequence of *cheA* gene (*XC_2284*). Tet^r^ | This work |

^a^Rif^r^, Kan^r^, Tet^r^, Ampr, Spc^r^ and Cat^r^ indicate resistance to rifampicin, kanamycin, tetracycline, ampicillin, spectinomycin and chloramphenicol, respectively.

**References**

Daniels, M.J., Barber, C.E., Turner, P.C., Sawczyc, M.K., Byrde, R.J.W., and Fielding, A.H. (1984) Cloning of genes involved in pathogenicity of *Xanthomonas campestris* pv. *campestris* using the broad host range cosmid pLAFR1. *EMBO J* **3:** 3323–3328.

Leong, S.A., Ditta, G.S., and Helinski, D.R. (1982) Heme biosynthesis in *Rhizobium*. Identification of a cloned gene coding for delta-aminolevulinic acid synthetase from *Rhizobium meliloti*. *J Biol Chem* **257:** 8724–8730.

Schäfer, A., Tauch, A., Jäger, W., Kalinowski, J., Thierbach, G., and Pühler, A. (1994) Small mobilizable multi-purpose cloning vectors derived from the *Escherichia coli* plasmids pK18 and pK19: selection of defined deletions in the chromosome of *Corynebacterium glutamicum*. *Gene* **145:** 69–73.

Staskawicz, B., Dahlbeck, D., Keen, N., and Napoli, C. (1987) Molecular characterization of cloned avirulence genes fromrace 0 and race 1 of *Pseudomonas syringae* pv. *glycinea*. *J Bacteriol* **169:** 5789–5794.

Yanisch-Perron, C., Vieira, J., and Messing, J. (1985) Improved M13 phage cloning vectors and host strains: nucleotide sequences of the M13mp18 and pUC19 vectors. *Gene* **33**: 103–119.
